# Prospects for advancing defense to cereal rusts through genetical genomics

**DOI:** 10.3389/fpls.2013.00117

**Published:** 2013-05-01

**Authors:** Elsa Ballini, Nick Lauter, Roger Wise

**Affiliations:** Corn Insects and Crop Genetics Research, Department of Plant Pathology and Microbiology, US Department of Agriculture - Agricultural Research Service, Center for Plant Responses to Environmental Stresses, Iowa State UniversityAmes, IA, USA

**Keywords:** eQTL, parallel expression, wheat, barley, *Puccinia*, *Triticeae*, cereal rusts

## Abstract

Rusts are one of the most severe threats to cereal crops because new pathogen races emerge regularly, resulting in infestations that lead to large yield losses. In 1999, a new race of stem rust, *Puccinia graminis* f. sp. *tritici* (*Pgt* TTKSK or Ug99), was discovered in Uganda. Most of the wheat and barley cultivars grown currently worldwide are susceptible to this new race. *Pgt* TTKSK has already spread northward into Iran and will likely spread eastward throughout the Indian subcontinent in the near future. This scenario is not unique to stem rust; new races of leaf rust (*Puccinia triticina*) and stripe rust (*Puccinia striiformis*) have also emerged recently. One strategy for countering the persistent adaptability of these pathogens is to stack complete- and partial-resistance genes, which requires significant breeding efforts in order to reduce deleterious effects of linkage drag. These varied resistance combinations are typically more difficult for the pathogen to defeat, since they would be predicted to apply lower selection pressure. Genetical genomics or expression Quantitative Trait Locus (eQTL) analysis enables the identification of regulatory loci that control the expression of many to hundreds of genes. Integrated deployment of these technologies coupled with efficient phenotyping offers significant potential to elucidate the regulatory nodes in genetic networks that orchestrate host defense responses. The focus of this review will be to present advances in genetical genomic experimental designs and analysis, particularly as they apply to the prospects for discovering partial disease resistance alleles in cereals.

## Introduction

The heteroecious rust fungi are some of the most important pathogens of cereal crops. These comprise 3000 *Puccinia* species (Van Der Merwe et al., 2007), including stem rust [*Puccinia graminis* f. sp. *tritici* (*Pgt*)], wheat leaf rust (*Puccinia triticina*), stripe rust (*Puccinia striiformis*), barley leaf rust (*Puccinia hordei*), and oat crown rust (*Puccinia coronata*). All of these species can infect a large range of cereal hosts: 365 grass species for *Puccinia graminis* alone.

Rust fungi pose a serious threat to cereal production because new races continue to emerge, and because infestation almost invariably leads to dramatic yield losses across large geographic areas (Leonard and Szabo, 2005; Bolton et al., 2008b). Breeding for genetic resistance to rusts reduces negative environmental impacts to agrosystems. In practice however, this approach is not infallible; the adaptability of rusts allows them to routinely overcome resistance gene (*R*) alleles bred into elite varieties (Singh et al., 2004a). Moreover, breeding efforts in response to new rust races does not always prevent crop loss, since wind dispersal of urediniospores can cause additional infections across continents in a short period of time (Hovmoller et al., 2008). Indeed, new races of leaf rust (Singh et al., 2004a), stripe rust (Milus et al., 2009), and stem rust (Stokstad, 2007) became widespread well before genetic resistance could be delivered in elite cultivars. Thus, the central challenge to the small grains industry is to reduce the periodicity of such outbreaks through renewed breeding efforts and continued management of epidemiological parameters that affect the evolution of pathogen virulence.

Stem rust, caused by the obligate fungal biotroph *Pgt*, has been a periodic, but serious problem wherever wheat and barley are grown (Roelfs, 1985; Leonard and Szabo, 2005). In North America, severe epidemics of stem rust have occurred from the late 1800's through the 1950's (http://www.globalrust.org). A new threat to wheat and barley production is the discovery of a novel race (*Pgt* TTKSK) of wheat stem rust in East Africa (Wanyera et al., 2006; Stokstad, 2007). This race, commonly called Ug99, is virulent to the majority of wheat varieties grown as well as advanced lines in current breeding programs (Jin and Singh, 2006; Bonman et al., 2007; Jin et al., 2007). *Pgt* TTKSK, and races of this lineage, infect barley as well as wheat, and has the potential to spread throughout the Middle East and to the Indian subcontinent in the near future (Singh et al., 2008). To overcome these new threats, additional resistances are needed in the short term, but more importantly, substantial new research efforts will be required in order to identify durable resistance to rusts over the long term (Ayliffe et al., 2008).

Genetic and/or molecular identification of novel sources of rust resistance in small grains will be greatly facilitated by recent gains in our basic knowledge of plant defense mechanisms. Plants detect the presence of the pathogen by two interconnected mechanisms (Jones and Dangl, 2006). One mechanism takes advantage of a specific response of the plant host induced by pathogen effectors; historically, these have has been referred to as gene-for-gene interactions (Flor, 1971), or in current terms, effector triggered immunity (ETI) (Jones and Dangl, 2006). This mechanism depends on direct or indirect recognition between pathogen effector (avirulence) proteins and plant R proteins (Innes, 2004; Deyoung and Innes, 2006; Deyoung et al., 2012). Another mechanism, designated PAMP triggered immunity (PTI), is induced by general elicitors or PAMPs (pathogen-associated-molecular-patterns), and is characterized by basal defense responses (Chisholm et al., 2006; Bent and Mackey, 2007). Based on this general doctrine, different strategies have been used to implement disease resistance in crops.

## Genetic strategies for deployment of host resistance in crops

The two primary genetic strategies for identification of disease resistance alleles useful for breeding are to focus on complete resistance conferred by *R* genes, or to focus on partial resistance that can be identified using quantitative genetic approaches. Significant progress has been made in identifying *R* genes in wheat and barley. More than 40 *R* genes that activate defense in response to *Puccinia triticina* and 40 *R* genes against *Pgt* have been mapped in wheat, as well as 20 *R* genes against *Puccinia striiformis* that have been mapped in wheat or barley (McIntosh et al., 1995; Ayliffe et al., 2008). Despite the dramatic success of these longstanding efforts, only a few *R* genes conferring resistance to a cereal rust have been cloned and functionally characterized: *Rpg1* (Brueggeman et al., 2002) and the *rpg4*/*Rpg5* complex (Brueggeman et al., 2008, 2009; Kleinhofs et al., 2009; Wang et al., 2013) in barley, *Rp1* (Collins et al., 1999) and *Rp3* (Webb et al., 2002) in maize, as well as *Lr21* (Huang et al., 2003) and *Lr10* (Feuillet et al., 2003) in wheat. While *R* genes tend to confer quite strong resistance to rusts in these cereal hosts, their efficacy in agronomic systems has the potential to be overcome by dynamic and rapidly evolving pathogen populations.

As such, a good strategy for countering the persistent adaptability of rusts is to deploy a combination of quantitative and qualitative resistance alleles. This strategy is typically more complicated to implement, but has the advantage of being more difficult to defeat, given that the various combinations are effective against a broader spectrum of races and thus, are believed to apply lower selection pressures (Singh et al., 2004b). Consequently, an agronomic phenotype often sought by breeders is non-specific partial resistance, or “slow rusting.”

Partial resistance loci are difficult to identify for three reasons. First, quantitative measurement of symptoms such as the length of latent period, pustule size and spore production require significant expertise and effort. Second, Quantitative trait locus (QTL) analyses aimed at identifying partial resistance loci require large population sizes in order to detect these less obvious effects (Singh et al., 2004b). Finally, specific combinations of alleles are often required in order for a partial resistance locus to display sufficient penetrance, which makes the parentage of the experimental populations critically important (Simmonds, 1988). Despite these challenges, two such partial resistance loci have recently been cloned, *Yr36* (Fu et al., 2009) and *Lr34* (Krattinger et al., 2009). These genes both define new classes of resistance genes, encoding a kinase with a putative START lipid-binding domain and an adenosine triphosphate-binding cassette transporter, respectively. Under field conditions, *Yr36* and *Lr34* confer quantitative levels of adult plant resistance and are expected to provide durable resistance to rusts in wheat. It should be noted that cloning these partial resistance genes required careful planning and large-scale execution of breeding strategies designed specifically for this type of effort (Simmonds, 1988).

## Molecular approaches to identify host defense genes and regulators

The identification of genes that have the capacity to confer quantitative levels of disease resistance to multiple pathogen races is an important step toward reliable crop protection over the long term (Poland et al., 2009). Although much of our understanding of PAMP- and ETI-mediated defense has been achieved through classic forward genetic approaches (Shirasu et al., 1999; Deyoung and Innes, 2006), there is great potential to combine these strategies with genome- or population-wide analysis of host transcriptomes during interactions with pathogens (Wise et al., 2007).

One of the exciting outcomes of these fundamental advances on host-pathogen interaction is the degree to which this basic knowledge is transferrable from one system to another. Regulators implicated in a specific interaction have been shown to play important roles in pathogen interactions across several species (Bent and Mackey, 2007; Shirasu, 2009). For example, *Rar1*, first identified in barley (Shirasu et al., 1999), has functional orthologs in Arabidopsis (Muskett et al., 2002), tobacco (Liu et al., 2002), rice (Thao et al., 2007), and wheat (Tai, 2008). Another example is the Arabidopsis PBS1 kinase, which is targeted by AvrPphB, a cysteine protease effector from *Pseudomonas syringae* pv. *phaseolicola* (Zhu et al., 2004). Cleavage of PBS1 by AvrPphB activates RPS5-specified resistance (Deyoung et al., 2012). PBS1 is widely conserved in monocots and dicots. Hence, a clear challenge is to accelerate discovery of such regulators, such that natural variants or transgenic alleles of these genes can be exploited (Innes, 2004; Deyoung and Innes, 2006; Shirasu, 2009; Deyoung et al., 2012).

Several cereal rust interactions have been investigated using parallel expression approaches (Table 1); not only to identify genes in particular defense pathways, but also to compare transcriptome reprogramming between mutants and their progenitors in order to clone defense regulators (Zhang et al., 2006) or genes involved in broad-spectrum resistance to stem rust (Zhang et al., 2008b). Upon comparison of the genes identified in these different experiments (Table 1), it was observed that the major classes of rust-responsive genes are similar with genes that are responsive during other biotic interactions. These include, but are not limited to genes that encode peroxidases, chitinases, pathogenesis related (PR) proteins, MAPK kinases, kinases, WRKY transcription factors, transport proteins, and proteins transported to chloroplasts. However, the regulation and the kinetics of expression for these genes may be vastly different, depending on the specific host-pathogen interaction that has been interrogated (Wise et al., 2007). A deeper comparative approach should facilitate discovery of common defense pathways between barley and wheat during interactions with rusts.

**Table 1:** **Investigations of differentially expressed genes or proteins during interaction between rust and cereal crops**.

**Host**	**Pathogen^a^**	**Isolate**	**Target gene (s)**	**Time^b^**	**Type^c^**	**References**
Barley	*Pgt*	TTKSK	*rpg4*, *Rpg5*	24	A	Moscou et al., 2011b
Barley	*Pgt*	MCC, QCC	*Rpg1, NecS1*	0, 6, 12, 18, 24, 36	A	Zhang et al., 2008a,b
Barley	*Pgt*	NI^d^	*Rpr1*	na	A	Zhang et al., 2006
Barley	*Ph*	1.2.1	*Rphq14*, *11*, *15*, and *8*	18	B	Chen et al., 2010a
Barley	*Ph*	1.2.1	*Rphq2*, *3*	18	B	Chen et al., 2010b
Barley	*Ph*	Dg2	*Rdg2a*	168, 336	C	Haegi et al., 2008
Wheat	*Pt, Ps*	MFBL	*Lr34*, *Yr18*	72	A	Hulbert et al., 2007
Wheat	*Pt*	BBB	*Lr1*, *Lr34*, *Tc*	72, 168	A	Bolton et al., 2008b
Wheat	*Ps*	PST-100 (06-194)	*Yr5*	6, 12, 24, 48	A	Coram et al., 2008a,c
Wheat	*Ps*	PST-78	*Yr39*	12, 24, 48	A	Coram et al., 2008b
Wheat	*Pt*	BBB, TJB	*Lr1*	3, 6, 12, 24	C	Fofana et al., 2007
Wheat	*Pt*	BBB	*Lr1*	24	D	Hu et al., 2007
Wheat	*Pt*	BBBD	*Lr1*	72, 144, 216	E	Rampitsch et al., 2006
Red fescue	*Puccinia* spp.	na	na	2, 8, 16, 24, 36, 48, 60	F	Ergen et al., 2007

a Pathogen abbreviation: Pgt, P. graminis f. sp. tritici (Stem Rust); Pt, P. triticina (Wheat Leaf Rust); Ps, P. striiformis (Stripe Rust); Ph, P. hordei (Barley Leaf Rust).

b Time, hours after inoculation.

c Type of technique used for data obtaining: A, Affymetrix GeneChip; B, Agilent oligonucleotide array; C, cDNA microarray; D, cDNA library; E, Proteomic; F, mRNA differential display.

d NI designates non-inoculated.

Approaches such as expression correlation and protein-protein interaction studies have facilitated the construction of defense pathways, but a clear understanding of the larger network of defense pathways and how they overlap remains elusive. The best documented defense network is based on work performed on *Arabidopsis thaliana* (Consortium, 2011; Mukhtar et al., 2011). Several reviews approach the defense response as a whole (Hammond-Kosack and Parker, 2003; Hofius et al., 2007), while others take a more focused approach (Mittler et al., 2004). Unfortunately, in cereals, graphical representations that efficiently foster a collective understanding are for the most part lacking, although several authors have reported pathway inferences from microarray experiments in rice (Cooper et al., 2003; Qiu et al., 2008). However, in these types of experiments, it is not possible to distinguish between cause and effect among correlated nodes. Several questions typically arise when co-expression networks are viewed: How can the key regulators of the pathway be identified? Are there connections between these regulators? Can the results of this network help a breeder make decisions? If so, what type of follow-up efforts should be pursued? Fortunately, a genetical genomic approach can provide the solution to these shortcomings. Surprisingly though, there has been little utilization of this approach in plant-pathogen interaction studies, despite overwhelming success when applied in human and animal disease research (de Koning and Haley, 2005; Mozhui et al., 2008).

## Genetical genomics offers new horizons to investigate plant defense mechanisms

QTL mapping finds statistical associations between genotypes and phenotypes, allowing regions of the genome harboring allelic differences that cause variation in the phenotype to be identified; these regions are called QTLs (reviewed by Mackay, 2001). Transcript abundance of a single gene is a quantitative trait and its regulation can be genetically interrogated. This is often called genetical genomics, or expression Quantitative Trait Locus (eQTL) mapping because the phenotypes in question are the expression of individual genes (Kendziorski and Wang, 2006; Rockman and Kruglyak, 2006). With the availability of high quality gene-expression platforms for barley (Close et al., 2004; Chen et al., 2010b), wheat (Schreiber et al., 2009), *Puccinia* spp. genome sequences (Cantu et al., 2011; Duplessis et al., 2011), as well as emerging next generation sequencing technologies (Li et al., 2010, 2013; Mayer et al., 2012), new strategies can be envisaged that interrogate both host and pathogen on a genome-wide, as well as a population-based scale.

The use of an eQTL strategy to identify and/or clone phenotypic QTL is well documented (Hansen et al., 2008). By profiling gene expression in each member of a segregating population, it is possible to use linkage analyses to identify key regulators of gene expression for a particular condition (Jansen and Nap, 2001; Rockman and Kruglyak, 2006; Williams et al., 2007; Kliebenstein, 2009; Li et al., 2013). For example, eQTL analysis of transcript abundance in embryo-derived tissues in barley has been combined with a QTL experiment on stem rust resistance in the same population (Druka et al., 2008). Three major QTL were detected in this population: two of them correspond to the known resistance genes *Rpg1* and *Rpg5/rpg4*, on chromosome 7H and 5H, respectively, and a third QTL was found on chromosome 2H. The cloned resistance gene *Rpg1* was detected as one of the best candidate genes to underlie the QTL detected on chromosome 7H, thereby substantiating the eQTL strategy for the candidate gene approach. Other strong candidates were detected for the two other loci. In another study, Moscou et al. (2011b) used an eQTL approach to identify a master regulator on chromosome 2H that controls hundreds of genes in response to Ug99 stem rust. In collaboration with U.S. and Kenyan partners, they showed that *rpg4*/*Rpg5*-mediated adult plant resistance is enhanced by allelic variants of the regulator. Thus, eQTL analysis is a useful strategy to identify and clone genes whose allelic variation results in phenotypic variation.

The eQTL strategy can also be applied to characterize gene networks or to confirm biological pathways (Keurentjes et al., 2007; Sonderby et al., 2007). For example, the Arabidopsis gene *ERECTA* is known to act pleiotropically on several pathways, including flowering time and resistance to bacterial wilt (Godiard et al., 2003). The role played by *ERECTA* in flowering time was confirmed using an eQTL mapping approach; in addition, new connections and regulatory nodes in *ERECTA*-specified pathways were identified (Keurentjes et al., 2007). Another interesting example is the MYB transcription factor MYB28, which was confirmed as a regulator of aliphatic glucosinolate, a defense metabolite in Brassicales (Sonderby et al., 2007). Several regulators have now been identified in plant/pathogen interactions. Network analysis should confirm the role played by these regulators, thus, developing a more complete picture of plant defense pathways.

Genetical genomics can also be used to investigate the heritability of gene expression, as well as the basis for transgressive segregation, where progeny phenotypes are statistically outside the range that would be predicted by parental phenotypes (Keurentjes et al., 2007; West et al., 2007; Li et al., 2013). Transgressive segregation has been measured in two plant studies (Keurentjes et al., 2007; West et al., 2007). In both cases ~50% of the genes show a significant difference in gene expression between parents and progeny in the population. This difference can be ascribed to the reassortment of additive genetic effects contributed by both parents, or to epistatic interactions among eQTL. Transgressive segregation is one of the reasons why some loci are difficult to use by breeders. Indeed, there are several cases of resistance genes that do not show the same phenotype in parental varieties as compared to their progenies. Understanding this phenomenon was required in order identify the suppressor of the *Lr34* leaf rust resistance gene in wheat (Vanegas et al., 2008). In another case, the *Lr13* leaf rust resistance gene is known to enhance resistance only in the presence of *Lr17* (Kolmer, 1992). The strength of eQTL analysis is that it provides the capacity to explain such complexities in a single experiment, rather than merely identifying the challenge.

## Challenges to designing, executing, and analyzing an eQTL experiment

Several recent reviews focus on the advances in genetical genomics (Doerge, 2002; Rockman and Kruglyak, 2006; Rosa et al., 2006; Williams et al., 2007; Wise et al., 2007; Gilad et al., 2008; Kliebenstein, 2009, 2010). To rapidly and efficiently discover partial or quantitative resistance alleles, one goal for future studies should be to understand how key regulators can modify gene expression, both temporally and spatially, during pathogen challenge and subsequent infection. In this section, we will outline parameters of experimental design to investigate this question, including factors that can influence the mapping process such as: population, experimental procedures and statistical analysis.

### Population types

Several factors determine the utility of a population for any given QTL mapping application, all of which are related to pedigree and/or population size (Lauter et al., 2008). Pedigree determines which alleles are contrasted, how many alleles per locus are tested, which modes of action can be investigated, and the degree of genetic resolution that is achievable. Population size partially determines genetic resolution and largely controls the level of statistical power that is available to accurately determine modes of allele action, including the detection of epistasis.

Most QTL studies in plants investigate allelic contrasts between only two alleles, which at some point in the pedigree were present in a single F_1_ plant. Common population types include F_2_, recombinant inbred line (RIL), intermated recombinant inbred lines (iRIL), double haploids (DHs), and back cross (BC). Based on simulation studies, a well-developed RIL population appears to be the most efficient for accurate QTL mapping (Ferreira et al., 2006). This makes sense, because during development of the population, each generation produces an additional round of meiotic recombination: i.e., *R* = 2*r* × (1 + 2*r*)^−1^, where *r* is the recombination frequency in the corresponding F_2_ (Burr and Burr, 1991). RIL populations, as well as DH and iRIL populations, also have the advantage of isogenicity, permitting experimental replication and testing of treatment effects without further genotyping.

A limitation of working with isogenic and true breeding lines is that the mode of allele action can not be determined, such that effects of recessive, dosage dependent, and dominant QTL alleles are indistinguishable without further analysis. This is a bigger limitation for breeding hybrid crops such as maize, where discovery of dominant QTL is preferred, than it is for inbred crops such as wheat or barley. Although limiting genotypic complexity is a drawback for allele characterization, it is experimentally beneficial in other regards. More statistical power to detect recessive and epistatic effects exists in a RIL population than in an F_2_ population of equivalent size. Imagine an extreme phenotype conditioned only by recessive allele action at three independent loci: in an F_2_ population, only one in 64 plants will have this genotype, compared to one in every eight plants in a RIL population. Detection of the main effects as well as the epistatic interdependence of these three hypothetical loci could only be revealed in an F_2_ study if a very large number of plants were used.

### Population size

Increasing the population size for a QTL experiment increases statistical power for both detection and localization of effects. Improved statistical power comes from larger numbers of lines or plants representing a particular genotype, while increased resolving power comes from additional recombination events that more closely flank the loci of interest. However, increased population size comes at a price. Thus, the challenge is to optimize population size as a function of price per significant gain in understanding the trait. Unfortunately, *a priori* determination of a minimum population size required for a particular level of success is largely an intuitive exercise. In combination with the population type, the mode of inheritance for the trait in question plays a prominent role in determining the genetic tractability of a trait. For example, a polygenic trait (in the classical sense) is much more difficult to dissect than one whose inheritance architecture is oligogenic (Lauter et al., 2004). For this reason, it is helpful to know the phenotypic distribution of the trait in order to have some indication of the underlying genotypic cause of something other than a normal phenotypic distribution.

### Are the genes determining resistance qualitative or quantitative?

A common case in plant pathological investigations, the presence of an effective *R* gene, illustrates this point quite clearly. It is common for partial resistance alleles to be more easily detected when they enhance the function of a resistance gene that has a major effect. There are many possible explanations for this, but a simple and intuitive way to look at it is that it is easier to distinguish between completely disease free and slightly diseased than between “mostly dead” and “all dead.” Suppose then that detection (for whatever reason) of a partial resistance allele epistatically depends on the presence of the *R* gene allele that confers resistance (Wise et al., 1996; Yu et al., 2001): in a RIL population, only half of the lines are useful for isolating the effect of this locus. The phenotypic distribution for such a trait should be strongly bimodal, which could be used to improve the experimental design prior to spending the money for genotyping. Further breeding and selection of a subset of lines for use are two of the simple solutions for this case.

### Optimizing populations

There have been some efforts to empirically determine the point of diminishing returns for manipulation of population size. Several simulation studies have predicted that a population of 200 RILs is required for a statistically accurate analysis (Kim et al., 2005; Silva et al., 2007). However, most of the time, such populations only allow the detection of phenotypic QTL with major effects, a severe limitation when partial resistance alleles are the target for discovery (de Koning and Haley, 2005). There are several useful tricks for overcoming the population size limitation without breaking the bank. One use of a genetical genomics approach is to identify key transcriptional regulators that likely harbor the genetic variation underlying phenotypic QTL. Accomplishing this feat requires good resolution of both the QTL and eQTL effects. The best way to globally improve genetic resolution without increasing population size is to intermate progenies during population construction. This breaks up linkage blocks without introducing additional alleles by capitalizing on successive rounds of recombination. The addition of four generations of intermating to a maize population breeding effort has been shown to provide up to 50-fold gains in genetic resolution (Balint-Kurti et al., 2007; Lauter et al., 2008). Another way to enhance resolution is to capitalize on evolutionary recombination events, as has been done with the nested association mapping (NAM) population for maize (Yu et al., 2008). However, the per-line gain in resolution of this approach is not yet clear. Moreover, the simultaneous use of many alleles in a partial resistance search is not advisable unless the nesting parent (B73 in the case of maize NAM) harbors an allele for which suppressors and enhancers are sought.

Increasing global resolution and power is often not the focus of an investigator's effort. Many pathologists already have ideal allelic contrasts ready for eQTL dissection with appropriate genetic materials, but simply wish to limit the effective population size in favor of improving replication. There are tricks for this as well. Potokina and associates (2008b) show that when the focus is on a particular phenotypic QTL, subsets of lines can be selected based on known recombination events and allelic composition to improve efficiency. To some degree, this strategy is generalizable any time an excess of previously genotyped lines exists (Rosa et al., 2006). Such selective phenotyping approaches sample the population in a way that minimizes segregation distortion while maximizing global recombination rate, thereby increasing both power and precision on a per line basis (de Koning et al., 2007). Indeed, the power of eQTL detection can be increased if a subset of the population is chosen for its genetic dissimilarity without a commensurate decrease in mapping power (Yan et al., 2006). Another appealing approach is to select two different subsets of the population for two different treatments (Li et al., 2008). In this way, it's possible to divide the population into two subsets with similar genetic background. During subsequent statistical analysis, the subset of the population used in one treatment (mock inoculated) can be considered as a reference for the other subset of the population used in the other treatment (inoculated), effectively doubling the number of lines compared to a classical experimental design; significantly increasing mapping power, as has been shown recently by Moscou and colleagues (2011b).

### Heritability

Since statistical power depends in part on heritability, the delicate balance of population size vs. replication needs to be optimized based on experimental goals. In general, when *de novo* detection of minor QTL effects is a primary goal, replication is much more important than when the aim is to more finely map a known minor effect locus. In our view, increasing population size in an eQTL experiment is its own form of replication, similar to how replicated evaluations of F_3_ families had previously been a norm for QTL mapping in plants (Cowen, 1988). The additional lines provide the benefit of new recombination events, while population measures such as mean and variance can be used to assess what proportion of total variance should be heritable. If a QTL allele cannot be reliably detected in a good experimental design, it is difficult to imagine how it will be of agronomic value.

Several wheat and barley populations have been used to map QTL for stem rust, leaf rust and stripe rust (Singh et al., 2004b; Druka et al., 2008; Chen et al., 2010a; Moscou et al., 2011b). However, small population sizes restrict the utility of these populations at present. A wider sampling of wheat and barley alleles would also provide stronger foundations for future research. Therefore, additional populations, preferably iRILs, should be created to allow discovery and fine mapping of new partial resistance loci. Particular attention must be paid to the *R* gene and QTL alleles carried when parents for these populations are selected.

### Treatment and statistical design for an eQTL experiment

Considering the statistical design details of an eQTL experiment, two principle questions should be resolved early: what alleles will be contrasted and under what conditions, where conditions need to include treatment, the tissue to be dissected, as well as the selection of the desired timepoint.

The scientific objectives generally pursued in molecular plant pathology are to find genes that capacitate critical steps in the specific interaction between host and pathogen. There are many possible experimental approaches available, including contrasts of host vs. non-host interactions, virulent vs. avirulent isolates, inoculated vs. mock-inoculated treatments, mutant vs. wild-type genotypes. Treatment design strongly affects the selection of alleles to be contrasted for pathological studies, as virulence vs. avirulence is so often controlled by gene-for-gene interactions between the pathogen and the host. Control treatments can also be beneficial, but are not necessarily required if the goal is to identify allelic differences that affect the regulation of transcription, rather than to characterize under what conditions the observed regulation occurs. In pathological experiments, non-inoculated control treatment often allow the researchers to distinguish between consequences of inoculation vs. infection, which can be essential for limiting the number of candidate genes that appear to regulate whether or not infection occurs (Moscou et al., 2011a,b).

In pathological experiments, timepoint needs to be considered as a function of plant development as well as a function of infection kinetics following controlled inoculation. It is important to know the kinetics of the interaction prior to designing an eQTL experiment, particularly if reducing costs is essential. The timing of events after inoculation have been measured for stem rust (Zhang et al., 2008a,b), leaf rust (Bolton et al., 2008a; Chen et al., 2010a), and stripe rust (Coram et al., 2008c). Penetration begins 12 hours after inoculation (HAI), haustorium formation at 18 HAI, and intercellular hyphal growth at 24 HAI (Sellam and Wilcoxson, 1976; Lin et al., 1998). The choice of timepoint within these parameters affects the outcome of the experiment. If resources were not limiting, an attractive option for a complete linkage and network analysis of infection would be to measure the expression of each individual of the population at each time point. In this way, one could track the eQTL actions as a temporal function of the interaction.

The tissue taken for RNA extraction should also be chosen carefully. Indeed, because only a small portion of the cells interact directly with the pathogen, the transcriptomic approach generally considers a population of cells, and thus, of an RNA mixture between interacting and non-interacting cells. For practical reasons, most microarray studies have used a seedling leaf 1- or 2-weeks old, with the assumption that the use of replicates would increase statistical power of small differences in expression. Yet, separation of even different leaf parts can be enlightening; in one study involving the interactions among wheat and wheat leaf rust and/or wheat stripe rust, significant differential gene expression was observed between basal and apical sections of each leaf (Hulbert et al., 2007).

Similarly, but not surprisingly, Potokina et al. (2008a) demonstrated that different eQTLs could be identified in embryo vs. seedling leaf tissue in barley. Wherever possible, one should collect tissue that is as narrowly defined as possible while minimizing perturbations to homeostasis (Li et al., 2010). In barley and wheat, epidermal peels are quite easy to isolate from the rest of the leaf; one can do so quickly and without wounding them such that the gain in specificity potentially outweighs the potential error introduced by manipulation. While this approach has proven useful for powdery mildew investigations (Zierold et al., 2005), no simple approach exists for rust research. Laser capture micro-dissection would be an option, but it would seem that this would be more appropriate if the experiment was focused on how infection and defense signaling are propagated across cell types.

### Challenges in statistical analysis

Several reviews have described methods for statistical analysis of microarray data and subsequent eQTL mapping procedures (Manly et al., 2004; Kendziorski and Wang, 2006; Williams et al., 2006; Jiang et al., 2008). First, microarray data are normalized in order to provide the phenotypic data for the expression level of each gene. If a linkage map already exists, eQTL mapping can commence immediately. Alternatively, microarray data can be used to create transcript-derived markers (TDMs) that can in turn be used to construct a genetic linkage map (Luo et al., 2007; Potokina et al., 2008b; Druka et al., 2010; Moscou et al., 2011b). This feature of eQTL mapping alone can often make the effort worthwhile; even with inexpensive genotyping, it is often cost effective in the long term to generate a TDM map of several thousand markers, which usually ensures that any two recombination events have a marker between them and that locations of genetic cross-overs are well-defined. TDMs can be integrated with Genotyping by Sequencing (GBS) approaches to anchor genetic maps to physical maps (Poland et al., 2012; Sonah et al., 2013).

Surprisingly, eQTL mapping is actually the simple portion of eQTL analysis work. The more difficult part is to figure out how to identify trends and meaningful patterns and in such a large volume of data, typically >10,000 regulatory relationships between an eQTL locus and the transcript it regulates. One of the most important things to establish is whether an eQTL acts in *cis* or *trans*, i.e., does the regulatory difference that leads to differential expression exist at the locus where the gene resides (*cis*) or elsewhere (*trans*). This level of characterization helps define the putative function of the eQTL, promoter difference vs. transcription factor difference, for example. Also, when searching for genes that act as capacitors of a significant process, it is helpful to know if many genes involved in a process are regulated by a locus where few or none of them reside. Such loci are termed eQTL hotspots and can regulate more than 1000 genes in some cases (West et al., 2007; Potokina et al., 2008a; Moscou et al., 2011b). Identification of eQTL hotspots is an effective way toward building gene networks, especially if one can identify a locus that regulates a cluster of genes associated with the biological phenomenon of interest, such as disease defense (Chen et al., 2010a; Moscou et al., 2011b). In barley and wheat, determining *cis* vs. *trans* is becoming clearer, with the recent emergence of genome sequence resources with many genes tied to a genetic and/or physical position (Brenchley et al., 2012; Mayer et al., 2012). Synteny coordinates in rice or Brachypodium can assist in such analyses. In addition, such an approach to hotspot detection can be enhanced by seeding the analysis only with genes that have at least two eQTL, which ensures that at least one of them must be acting in *trans*.

Beyond the many possible methods to identify eQTL hotspots (Williams et al., 2007), the nature of their action remains unclear: could they be gene-dense regions where recombination is limited, or do they actually exist as the signature of a high-level regulatory gene (Breitling et al., 2008)? Notably, the statistical models described to date do not consider the influence of gene function, co-location patterns or co-expression. Plant resistance and defense genes are often clustered in the genome; the influence of such genomic organization on eQTL detection needs to be integrated into the statistical analysis. Indeed, simulations have shown that expression correlation can explain a large part of eQTL co-localization (Wang et al., 2007). Fortunately, permutation tests should be able to assess the reliability of putative eQTL hotspots (Breitling et al., 2008).

Some of the statistical challenges could also be viewed as opportunities. When it is not possible to determine which force is acting, it should be true that tracking of either phenomena can lead to the source. For example, co-location of several hotspots that regulate genes with correlated expression patterns could lead to more robust eQTL detection. Indeed, within an eQTL hotspot, one of the best candidates for the “master regulator” may be a gene whose expression correlates with the other genes whose eQTL have mapped to the same locus. This parsimonious inference has been applied to further test the predicted flowering time network in Arabidopsis (Keurentjes et al., 2007) and also between particular eQTL and transcription factors in yeast (Sun et al., 2007). For plant pathological applications, many transcription factors regulating defense are known, facilitating application of this approach.

### Epistasis

Epistasis has been shown to have an impact on numerous major phenotypic QTL and will likely explain significant variance components of plant gene expression (Rowe and Kliebenstein, 2008). However, the epistatic interdependencies of gene expression have generally been neglected in plant eQTL analysis studies (Sun et al., 2008). While such tests require more statistical power than is usually available, future populations and experimental designs are sure to be more powerful, so a challenge to build the analysis infrastructure and to improve definition of the statistical underpinnings for large-scale tests for epistasis lie clearly before us. It will be necessary to define common criteria and performance measures for such analyses in order to permit the establishment of a collective intuition that is meaningful for evaluation of inferences. To this end, the routine sharing of data and analysis methods in a database such as PLEXdb (http://plexdb.org/) (Dash et al., 2012), WebQTL, or GeneNetwork (http://www.genenetwork.org/) facilitates these goals.

## Concluding remarks

Genetical genomics offers a new approach to the study of plant pathogen interactions. This systems biology approach leverages the complementary strengths of classical genetics and transcriptomics to connect loci that confer resistance with gene expression networks that are responsive to infection. Given these complementary strengths, it is incumbent upon the community to have the vision to perform definitive experiments to associate cause and effect. In this way, these experiments will facilitate the identification and cloning of new loci as well as known phenotypic QTL.

For the near term, the community has only some of these resources in hand, so the challenge is to carry out beneficial experiments with existing resources, while continuing to develop the next generation of tools to answer critical questions. These include: To what extent do polymorphisms in transcription factors and the promoter regions with which they interact govern the outcome of plant defense? What has the highest influence on gene regulation: polymorphism in regulators, polymorphism in downstream pathway, or variability in the environment? What is the evolution of defense regulators and how are they maintained in populations?

Suggested experiments to begin to answer these questions should connect kinetics of pathogen infection with responsive host genes and regulatory networks. In order to best track individual eQTL through the interaction, an optimal experimental plan would require: (1) high resolution population(s) that harbor genetic variation for resistance to the pathogen—intermated RILs should provide the resolution, while simultaneously providing a reasonable number of individuals for downstream molecular work; (2) all-genes expression-profiling platforms for the hosts and pathogens in question—with NextGen sequencing technologies, these are becoming possible at a reasonable cost; (3) high-throughput genotyping—several platforms offer the possibility of genotyping individuals with multiplex capability (Poland et al., 2012; Sonah et al., 2013), and (4) detailed infection phenotyping—with a reasonable number of intermated RILs, response to multiple pathogens or isolates could realistically be accomplished. In addition to these host parameters, one could dramatically increase the power of the investigation if equivalent resources (population, expression profiling, genotyping) were in place for the pathogen. In that case, exploration of “all by all” (segregating host by segregating pathogen) could be pursued.

### Conflict of interest statement

The authors declare that the research was conducted in the absence of any commercial or financial relationships that could be construed as a potential conflict of interest.

## Glossary of definitions

Partial resistance is a form of resistance, phenotypically characterized by a temporally and/or spatially reduced rate of pathogen development on the host (Parlevliet, 1978); this also may be referred to as Quantitative resistance (Poland et al., 2009). Genetical Genomics refers to the use of quantitative genetic mapping to dissect the regulatory underpinnings of molecular phenotypes collected *en masse* using high-throughput genomic technologies (Jansen and Nap, 2001). expression Quantitative Trait Locus (eQTL) mapping is the treatment of transcript abundance as a quantitative trait.

## Abbreviations

eQTL: (gene) expression quantitative trait locus
PAMP: pathogen-associated-molecular-pattern
ETI: effector triggered immunity
PTI: PAMP triggered immunity
DH: doubled haploid
RIL: recombinant inbred line
TDM: transcript-derived marker.
